# The Role of Peritoneal Dialysis in the Treatment of Acute Kidney Injury in Patients With Acute-on-Chronic Liver Failure: A Prospective Brazilian Study

**DOI:** 10.3389/fmed.2021.713160

**Published:** 2021-09-23

**Authors:** Daniela Ponce, Welder Zamoner, Dayana Bitencourt Dias, Erica Pires da Rocha, Christiane Kojima, André Luís Balbi

**Affiliations:** ^1^Department of Internal Medicine, Botucatu Medical School – University of São Paulo State – UNESP, São Paulo, Brazil; ^2^Botucatu Medical School – University of São Paulo State – UNESP, São Paulo, Brazil

**Keywords:** liver cirrhosis, acute-on-chronic liver disease, acute kidney injury, peritoneal dialysis, mortality

## Abstract

This study aimed to explore the role of peritoneal dialysis (PD) in acute-on-chronic liver disease (ACLD) in relation to metabolic and fluid control and outcome. Fifty-three patients were treated by PD (prescribed Kt/V = 0.40/session), with a flexible catheter, tidal modality, using a cycler and lactate as a buffer. The mean age was 64.8 ± 13.4 years, model of end stage liver disease (MELD) was 31 ± 6, 58.5% were in the intensive care unit, 58.5% needed intravenous inotropic agents including terlipressin, 69.5% were on mechanical ventilation, alcoholic liver disease was the main cause of cirrhosis and the main dialysis indications were uremia and hypervolemia. Blood urea and creatinine levels stabilized after four sessions at around 50 and 2.5 mg/dL, respectively. Negative fluid balance (FB) and ultrafiltration (UF) increased progressively and stabilized around 3.0 L and −2.7 L/day, respectively. Weekly-delivered Kt/V was 2.7 ± 0.37, and 71.7% of patients died. Five factors met the criteria for inclusion in the multivariable analysis. Logistic regression identified as risk factors associated with Acute Kidney Injury (AKI) in ACLD patients: MELD (OR = 1.14, CI 95% = 1.09–2.16, *p* = 0.001), nephrotoxic AKI (OR = 0.79, CI 95% = 0.61–0.93, *p* = 0.02), mechanical ventilation (OR = 1.49, CI 95% = 1.14–2.97, *p* < 0.001), and positive fluid balance (FB) after two PD sessions (OR = 1.08, CI 95% = 1.03–1.91, *p* = 0.007). These factors were significantly associated with death. In conclusion, our study suggests that careful prescription may contribute to providing adequate treatment for most Acute-on-Chronic Liver Failure (ACLF) patients without contraindications for PD use, allowing adequate metabolic and fluid control, with no increase in the number of infectious or mechanical complications. MELD, mechanical complications and FB were factors associated with mortality, while nephrotoxic AKI was a protective factor. Further studies are needed to better investigate the role of PD in ACLF patients with AKI.

## Introduction

Acute kidney injury (AKI) is a common complication of acute-on-chronic liver failure (ACLF), occurring in up to 20% of hospitalized cirrhotic patients (1). The main reasons for the development of AKI in patients with decompensated cirrhosis are infections, hypovolemia associated with bleeding or the use of diuretics, nephrotoxicity (drug-induced or contrast-induced nephropathy), hepatorenal syndrome (HRS), and parenchymal nephropathy (2–4).

A large study of 463 hospitalized ACLF patients with AKI evaluated the frequency and prognosis of the different etiologies of AKI. This study demonstrated that the most frequent cause of AKI among cirrhotic patients was bacterial infection (46%), followed by volume depletion (32%), HRS (13%), and parenchymal nephropathy (9%). Among infections, spontaneous bacterial peritonitis (SBP) and spontaneous bacteremia were the most common. The 90-day mortality was high (60%), but it was particularly high among patients with AKI associated with infections or HRS (2) and among patients that needed kidney replacement therapy (KRT), reaching 80% (2–5).

KRT may be considered as a rescue therapy for patients with decompensated end-stage liver disease, for whom pharmacological treatment is ineffective and there are no contraindications for liver transplantation (6). The indication for KRT follows standard guidelines and is not specific to patients with cirrhosis and AKI. Conventional indications include volume overload not responding to diuretics, uremia, metabolic acidosis and refractory hyperkalemia. In patients with HRS, KRT should be indicated in the absence of response or adverse reaction to vasoconstrictors (5, 6).

Liver transplantation is the only treatment modality for the reversal of AKI associated with HRS (HRS-AKI) in the cirrhotic setting, while KRT is a bridging therapy aimed at keeping the patient alive until receiving the graft (6–9). The assessment of prognosis, eligibility for liver transplantation, and advanced stages of cirrhosis should be considered before KRT to avoid futile treatments (7–9).

Acceptable KRT methods are intermittent (iHD) or prolonged HD (PHD), continuous haemofiltration or continuous haemodiafiltration (CRRT), and peritoneal dialysis (PD). The choice of the dialytic method is critical in decompensated cirrhotic patients. Hypotensive reactions and blood clotting abnormalities are more frequent during haemodialysis (HD) in cirrhotic patients than in patients with an intact liver. The most important limiting factor of intermittent HD is haemodynamic instability and PHD; CRRT and PD may be better tolerated (8, 10, 11). PD is also able to remove ascites fluid, does not increase the number of complications, and does not expose patients to anticoagulants (12).

Given the paucity of evidence in this important area, the aim of this study was first to investigate the in-hospital mortality of ACLF patients treated using PD; second, to determine the metabolic and fluid control, and third to identify the risk factors associated with death.

## Methods

### Study Population

This study is a sub-analysis of a larger retrospective observational study that investigated the epidemiology of AKI and its effect on patient outcomes across time periods (13). This study was separately approved by the Ethics Committee of Botucatu School of Medicine University Hospital, Sao Paulo, Brazil (protocol 30457414.7.0000.5411). Written informed consent was obtained from all patients or relatives prior to their inclusion in the study. Decompensated cirrhotic patients who had been consecutively treated by HVPD were evaluated between July 2012 and June 2020.

Patients who were hospitalized with ACLF as the primary diagnosis and had ischemic or nephrotoxic stage 3 AKI according to the KDIGO criteria were eligible for enrolment (14). Definitions of ACLF and the hepatorenal syndrome type of AKI were based on those from the American Association for the Study of Liver Diseases and European Association for the Study of the Liver (15). Indications for dialysis were uremia or azotemia (blood urea nitrogen [BUN] >100 mg/dL), hypervolemia (after diuretic use), electrolyte imbalance (K >6.5 mEq/L after clinical treatment), and acid-base disturbance (pH <7.1 and bicarbonate <10 mEq/L after clinical treatment) (13–15). Exclusion criteria were age under 18 years, advanced chronic kidney disease (CKD) [glomerular filtration rate lower than 30 mL/min, using the baseline creatinine and CKD-EPI formula (16)], renal transplantation, pregnancy, other etiologies of AKI (post-renal and glomerulonephritis), and absolute contraindication for PD (recent abdominal surgery [<1 month], multiple abdominal surgeries [>3], severe hyperkalemia with electrocardiogram [EKG] changes, severe respiratory failure [fraction of inspired oxygen (FiO_2_) >70%], acute pulmonary oedema) (17–19). Acute pulmonary oedema was diagnosed based on the patient's history (abrupt onset), clinical examination (orthopnea, severe respiratory distress, and rales and crackles over lungs), chest X ray (diffuse alveolar or interstitial oedema), and oxygen desaturation (<90% in room air).

If patients presented any one of these contraindications, they were treated by intermittent conventional or prolonged HD according to their haemodynamic instability.

### Study Protocol

The PD was performed according to previous studies by Ponce et al. and the ISPD guidelines for peritoneal dialysis in AKI: 2020 update (17–20). The prescribed Kt/V was 0.4/session, total volume ranged from 18 to 32 L/day, and a tidal modality was used to avoid the removal of all ascites fluid. Peritoneal access was established by blind percutaneous placement of a flexible catheter using the Seldinger technique. Cephazolin was used as a prophylactic antibiotic to cover PD catheter insertion. Patients were treated with continuous PD and exchanges with Dianeal PD solution (Na = 135 mEq/L, Ca = 3.5 mEq/L, *K* = 0 mEq/L, Mg = 1.5 mEq/L, lactate = 40 mEq/L, 1.5–2.5% dextrose) were performed using a HOMECHOICE cycler. To evaluate the adequacy of the dialysis, delivered Kt/V, ultrafiltration (UF), and fluid balance (FB) were calculated daily.

Baseline body weight was recorded at initial hospital admission. Oliguria was defined as urine output <0.5 mL/kg/h for at least 6 h. The cumulative FB was registered 48 h before starting dialysis. To quantify the cumulative FB over 2 days in relation to body weight, we used the following formula: sum of daily (fluid intake [L] – total output [L])/body weight (kg). We used the term percentage of fluid accumulation to define the percentage of cumulative FB adjusted for body weight. We defined fluid overload (FO) as fluid accumulation >5% of the baseline weight (21).

Other variables, including comorbidity, laboratory investigations, urine output, number of dialysis sessions, need for mechanical ventilation, presence of haemodynamic instability, model of end stage liver disease (MELD), duration of hospitalization, and causes of mortality were analyzed. Thereafter, ACLF patients treated with HVPD were divided into two groups (survival and no survival) and compared.

The protocol was interrupted when there was partial recovery in renal function (urine output >1,000 mL/day and progressive drop in creatinine [<4 mg/dL] and BUN levels [<50 mg/dL]), a need to change dialysis method because of infectious or mechanical complications, failure of HVPD to remove fluid and solute, more than 28 days of follow-up, or death.

### Statistical Analysis

Results are presented as mean and standard deviation or median, according to normality characteristics for each variable. Student's *t*-test was used to compare parametric variables between two groups and the Mann-Whitney test was used for non-parametric variables. Categorical variables were expressed as proportions and compared with the chi-square or Fisher's exact test. Variables with significant univariate associations were considered as candidates for multivariable analysis. Longitudinal multivariable logistic regression was performed using backward variable selection, with the exit criteria set at *p* < 0.25. Variables not selected by the automated procedure were added back into the models individually to evaluate residual confounding and covariance, and we tested for colinearity among all variables using univariate analysis to identify possible associated confounding variables. Subsequently, through the construction of a logistic regression model, multivariate analysis was performed with odds ratio (OR) calculations, including all independent variables that showed association with the mortality, with *p* ≤ 0.20.

All statistical analyses were performed on an intention-to-treat basis using SPSS 17.0 for Windows statistical software (SPSS, Chicago, IL, USA), with a two-sided *p* < 0.05 considered to be statistically significant.

## Results

During the study period (8 years), a total of 132 ACLF patients were treated by dialysis: 53 by PD (40.1%) and 79 by HD (59.9%), of which 35 were treated by conventional and 44 by prolonged HD. Patients treated using HD had relative contraindications for PD use. The main of them were spontaneous bacterial peritonitis (51%) and need for an inspired oxygen fraction >70% (36.7%). Generally, in our hospital, we use PD primarily as a treatment option for AKI patients who do not present contraindications for this method.

The mean age was 64.8 ± 13.4 years, 38 patients were male (71.7%), 52.8% were Caucasian, 20.7% had hypertension, and 24.5% had diabetes mellitus. MELD at hospital admission was 31 ± 6 and the patient mean weight was 59.6 ± 6.5 kg; 58.4% of weight measurements were obtained by digital scale, 16.9% by bed scale and 24.6% were calculated from two-variable formulas. Most patients (58.5%) were in the intensive care unit (ICU) and 41.5% were in the wards. A total of 77.4% of the patients had been hospitalized for decompensated cirrhosis during the previous year. Ischaemic acute tubular necrosis (iATN) was the most common cause of AKI (60.4%), followed by nephrotoxic ATN AKI (24.5%), and hepatorenal syndrome (HRS) (18.0%).

Alcoholic liver disease was the main cause of cirrhosis HF (50.9%), followed by viral hepatitis B and C (28.3%), and non-alcoholic fatty liver disease (20.8%). The main precipitating causes of decompensation were infection or sepsis (37, 69.8%) and variceal bleeding (13, 24.5%).

Before indication for dialysis, all patients received at least 1 mg/kg of intravenous furosemide twice a day, 58.5% needed intravenous inotropic agents including terlipressin, and 69.8% were on mechanical ventilation. The median number of HVPD sessions was 5 (4–8, 10).

Table 1 shows the improvement of metabolic control and FB after PD initiation. Blood urea nitrogen and creatinine levels stabilized after 4 sessions and bicarbonate and pH levels after 3 sessions. The mean UF increased steadily from 1 to 3 sessions and stabilized after 4 sessions at around 2.6 L/day. There was a progressive increase in negative FB from 1 to 3 HVPD sessions, with FB stabilization after 3 sessions at around −2.7 L/day. Table 2 shows the prescribed and delivered dialysis dose parameters. The delivered urea Kt/V was 0.38 ± 0.06 per session and 2.7 ± 0.37/week.

**Table 1:** Serum BUN, creatinine (Cr), bicarbonate (Bic), pH, potassium (K), ultrafiltration (UF), and fluid balance (FB) at the beginning of treatment and after each session of peritoneal dialysis in acute-on-chronic liver failure patients.

	**Sessions**						
	**Pré**	**1**	**2**	**3**	**4**	**5**	**6**
	***N* = 53**	***N* = 52**	***N* = 50**	***N* = 41**	***N* = 32**	***N* = 20**	***N* = 15**
BUN (mg/dl)	101 ± 31	89 ± 28	79 ± 22	63 ± 19	51 ± 14	48 ± 12	46 ± 10
Cr (mg/dl)	3.1 ± 1.2	3.2 ± 1.4	2.8 ± 0.8	2.4 ± 0.8	2.1 ± 0.9	2.3 ± 0.7	2.2 ± 0.6
Bic (mEq/L)	16.8 ± 3.7	17.4 ± 4.2	21.9 ± 4.2	22.7 ± 3.6	22.5 ± 3.8	23.1 ± 3.4	23.2 ± 3.2
pH	7.24 ± 0.1	7.28 ± 0.1	7.31 ± 0.2	7.32 ± 0.2	7.31 ± 0.2	7.33 ± 0.3	7.34 ± 0.2
K	5.4 ± 0.8	4.7 ± 0.7	4.2 ± 0.4	4.0 ± 0.3	3.8 ± 0.3	4.0 ± 0.3	3.8 ± 0.2
UF (l/d)	–	2.1 ± 0.8	2.9 ± 0.9	2.8 ± 0.9	2.7 ± 0.8	2.9 ± 0.7	2.8 ± 0.8
FB (l/day)	5.8 ± 1.9	−0.7 ± 0.4	−1.9 ± 0.9	−2.2 ± 1.7	−2.3 ± 0.8	−2.1 ± 0.7	−2.3 ± 0.7
Lactate (mmol)	2.4 ± 0.8	2.9 ± 1.1	2.74 ± 0.9	2.6 ± 0.9	2.5 ± 0.7	2.8 ± 0.9	2.7 ± 0.8
Sodium (mEq/L)	128 ± 11	129 ± 09	131 ± 11	130 ± 10	133 ± 08	132 ± 09	131 ± 07
Platelet count (/mm^3^)	65.230 ± 13.120	61.720 ± 12.170	62.350 ± 10.962	59.310 ± 9.460	62.770 ± 12.580	61.180 ± 10.470	64.219 ± 10.116

*BUN, blood urea nitrogen; Cr, creatinine; Bic, bicarbonate; K, potassium; UF, ultrafiltration; FB, fluid balance*.

**Table 2:** Peritoneal dialysis prescription and adequacy.

**Variables**	
Dialysate volume per cycle (ml)	30 ml/kg (1,500–2,100 ml)
Inflow time (min)	10
Dwell time (min)	50–90
Outflow time (min)	20
Cycle duration (min)	80–120
Total exchanges per session	10–20
Session duration (h)	24
Total dialysate volume per session (l)	18–32
% glucose	1.5–2.5
**Prescribed Kt/V**	
Per session	0.4
Weekly	2.8
**Delivered Kt/V**	
Per session	0.38 ± 0.06^a^
Weekly	2.7 ± 0.37^a^

a *Without significant difference from prescribed Kt/V*.

Peritonitis related to PD occurred in six patients (11.3%) after 9.6 ± 2.3 PD sessions. Four patients (66.7%) had the catheter removed and the dialysis method changed because of no improvement in laboratory or clinical parameters after 5 days of correct antibiotic treatment. The main etiologic agents were *Pseudomonas aeruginosa* or fungi. Antibiotic treatment was maintained from 14 to 21 days.

Only four patients presented mechanical complications (7.6%), with leakage being the most frequent (75%), without need for interruption of therapy. The dialysate volume per cycle was reduced from 30 to 20 ml/kg per cycle (around 1,200 mL/cycle). Only one patients had wound bleeding.

**Figure 1:**
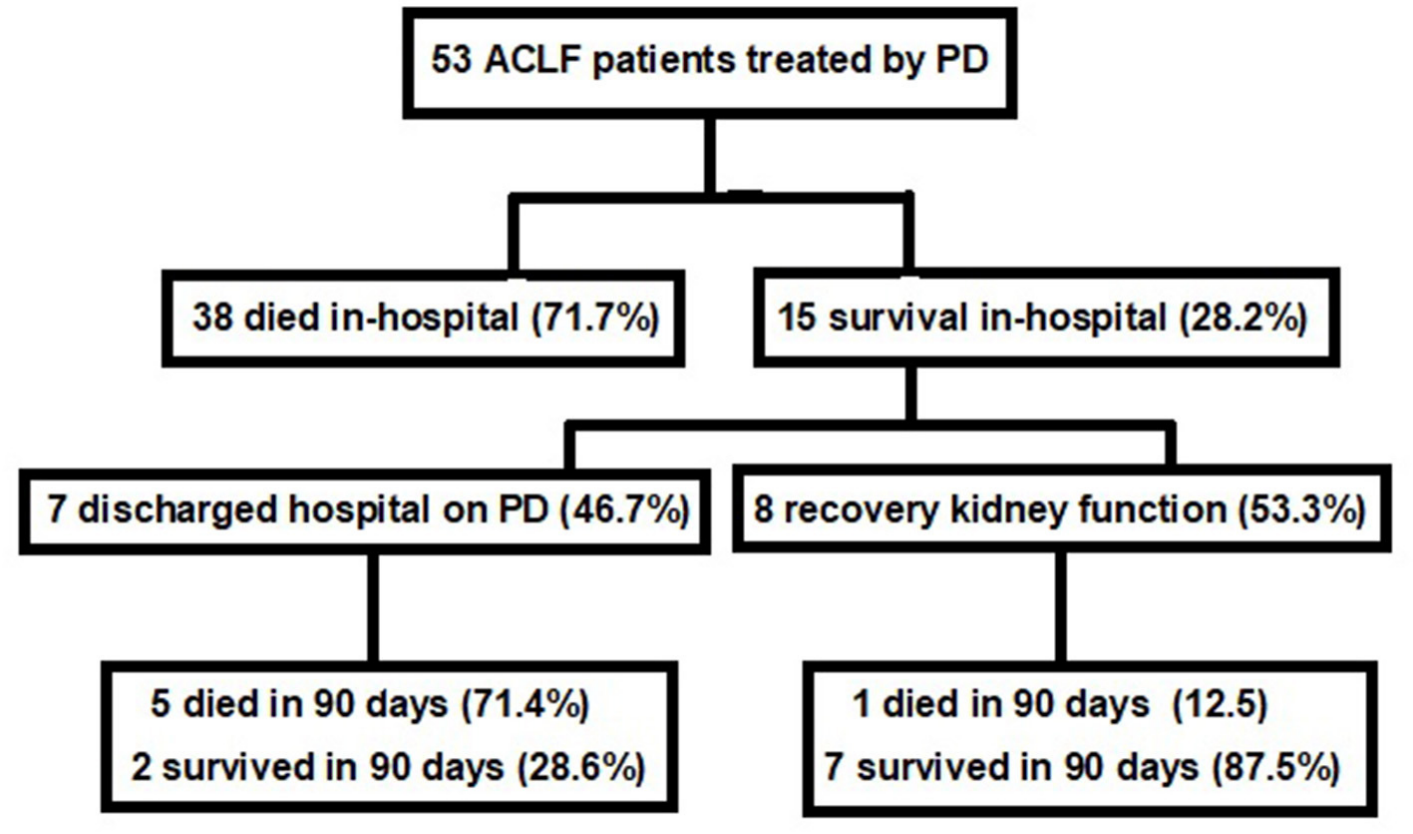
Acute-on-chronic liver failure patients outcome treated with peritoneal dialysis.

Change in the dialysis method occurred in three patients (5.7%) due to refractory peritonitis. Concerning patient outcome, eight (15.1%) recovered renal function, while seven patients (13.2%) were kept on dialysis after hospital discharge. In-hospital mortality was 71.7% (9) and the main cause of death was sepsis (81.6%). Among the survivors, recovery of kidney function was 53.3% at hospital discharge. After 90 days, only nine patients were alive (16.9%). Figure 1 shows the acute-on-chronic liver failure patients outcome treated with PD.

Survivors (S) and non-survivors (NS) in-hospital were similar in gender, cause of cirrhosis and vasoactive drug use. Azotemia was the main indication for dialysis in both groups. There was no difference in metabolic control between the S and NS patients. The groups had similar values of delivered Kt/V per session and weekly (NS: 0.36 ± 0.11 vs. S: 0.38 ± 0.12, *p* = 0.57, and NS: 2.52 ± 0.7 vs. S: 2.66 ± 0.8, *p* = 0.58) and rate of infectious and mechanical complications related to PD (NS: 10.5 vs. S: 6.9%, *p* = 0.81, and NS: 7.9 vs. S: 6.7%, *p* = 0.68).

There was a difference between the groups in terms of age (NS: 67.4 ± 11.7 vs. S: 60.6 ± 10.6, *p* < 0.001), MELD (NS: 32 ± 11 vs. S: 21 ± 8, *p* < 0.001), nephrotoxic ATN as the cause of AKI (NS: 7.9% vs. S: 53.3%, *p* < 0.001), mechanical ventilation (NS: 84.2 vs. S: 33.3%, *p* < 0.001), urine output at day of dialysis indication (NS: 348 ± 77 vs. S: 888 ± 252 mL), and number of PD sessions [NS: 4 (3–6) vs. S: 7 (4–9)], FO and UF from the 2nd to 4th PD sessions (Tables 3, 4). Survivors presented higher UF and lower FB than non-survivors.

**Table 3:** Acute-on-chronic liver failure patients distribution treated with peritoneal dialysis according to outcome and main clinical and laboratory characteristics.

	**General (*n* = 53)**	**No-survival (*n* =38)**	**Survival (*n* = 15)**	***P***
**Age (years)**	64.8 ± 13.4	67.4 ± 11.7	60.6 ± 10.6	0.001
**Male sex (%)**	38 (71.7)	27 (71.1)	11 (73.3)	0.97
**Weight (kg)**	59.6 ± 6.5	57.6 ± 6.1	61.4 ± 6.7	0.08
**Diabetes (%)**	13 (24.5)	9 (23.1)	4 (26.6)	0.96
**Dialysis indication**				
Azotemia-uremia (%)	38 (71.7)	28 (73.7)	10 (66.7)	0.94
Hyperkalemia (%)	8 (15.1)	4 (10.6)	4 (26.7)	0.21
Hypervolemia (%)	5 (9.4)	4 (10.5)	1 (6.7)	0.61
Others^*^ (%)	2 (3.7)	2 (5.3)	0 (0)	0.82
**AKI etiology (%)**				
Ischaemic AKI	32 (60.4)	28 (73.7)	4 (26.6)	0.005
Nephrotoxic AKI	11 (20.8)	3 (7.9)	8 (53.3)	<0.001
HRS AKI	10 (18.9)	7 (18.4)	3 (20)	0.98
**Cause of cirrhosis (%)**				
Alcoholic liver disease	27 (50.9)	18 (47.4)	9 (60)	0.61
Viral hepatitis	15 (28.3)	10 (26.3)	5 (33.3)	0.73
Non-alcoholic fatty liver disease	11 (20.8)	10 (26.3)	1 (6.7)	0.14
**Causes of precipitating decompensation (%)**				
Variceal bleeding	13 (24.5)	8 (21)	5 (33)	0.48
Infectious or sepsis	37 (69.8)	28 (73.7)	9 (60)	0.97
**MELD**	31 ± 6	33.8 ± 11.2	24.9 ± 8.5	<0.001
**FO pre dialyis (%)**	22 (41.4)	16 (42.1)	6 (40)	0.86
**Mechanical ventilation (%)**	37 (69.8)	32 (84.2)	5 (33.3)	0.003
**Vasoactive drugs (%)**	31 (58.5)	25 (65.8)	6 (40)	0.15
**Urine output (ml)**	582 ± 161	348 ± 77	888 ± 252	0.04
**PD complications (%)**				
Peritonitis	6 (11.3)	4 (10.5)	2 (13.3)	0.92
Mechanical (leakage)	4 (7.6)	3 (7.9)	1 (6.7)	0.97
**Number of sessions (days)**	5 (4–9)	4 (3–6)	7 (4–9)	0.03

*AKI, acute kidney injury; FO, fluid overload; HRS, hepatorenal syndrome; MELD, model of end stage liver disease;*

* *Others, acidosis, more than one indication; PD, peritoneal dialysis*.

**Table 4:** Acute-on-chronic liver failure patients distribution treated with high volume peritoneal dialysis according to outcome and metabolic control.

	**No-survival (*n* = 38)**	**Survival (*n*= 15)**	***P***
**Pre BUN (mg dl)**	103.7 ± 28.3	109.7 ± 39.9	0.57
**Pre creatinine (mg dl)**	5.2 ± 2.8	5.4 ± 2.3	0.79
**BUN after (mg dl)**			
1st session	95 ± 42	89 ± 32	0.34
2nd session	86 ± 31	79 ± 21	0.29
3rd session	71 ± 27	68 ± 19	0.39
4th session	62 ± 18	51 ± 22	0.41
5th session	54 ± 15	49 ± 11	0.57
**Creatinine after (mg dl)**			
1st session	5.1 ± 1.5	5.4 ± 1.6	0.64
2nd session	4.6 ± 1.2	4.9 ± 1.4	0.61
3rd session	4.3 ± 1.3	4.7 ± 1.5	0.76
4th session	3.9 ± 1.1	4.2 ± 1.3	0.47
5th session	3.7 ± 1.1	4.1 ± 1.2	0.71
**Bicarbonate after (mg dl)**			
1st session	16.4 ± 4.7	18.1 ± 4.9	0.54
2nd session	20.1 ± 4.3	21.1 ± 4.7	0.59
3rd session	21.2 ± 3.5	21.9 ± 4.5	0.69
4th session	22.5 ± 3.4	22.8 ± 4.4	0.71
5th session	22.8 ± 3.1	23.7 ± 4.1	0.77
**UF after**			
1st session	0.83 (−0.33 to 1.5)	1.3 (−0.8 to 1.5)	0.14
2nd session	1.9 (0.9–2.8)	2.8 (0.9–3.3)	0.05
3rd session	1.4 (0.8–2.3)	2.9 (1.0–3.7)	0.04
4th session	2.1 (0.8–2.4)	2.9 (1.4–3.4)	0.06
5th session	2.4 (0.9–1.6)	2.8 (1.9–3.3)	0.11
**FB after**			
1st session	1.22 ± 0.4	0.8 ± 0.1	0.37
2nd session	−1.69 ± 0.4	−2.88 ± 0.7	0.04
3rd session	−1.96 ± 0.9	−3.15 ± 0.1	0.03
4th session	−2.15 ± 1.1	−3.19 ± 1.1	0.04
5th session	−2.51 ± 0.8	−2.92 ± 0.9	0.09
**Delivered Kt/ V**			
Per session	0.36 ± 0.11	0.38 ± 0.12	0.57
Weekly	2.52 ± 0.7	2.66 ± 0.8	0.58

*After the second PD session.*

*FO, fluid overload; FB, fluid balance*.

Five factors met the criteria for inclusion in the multivariable analysis: age, nephrotoxic AKI, mechanical ventilation, MELD, and FO after two sessions. MELD (OR = 1.14, CI 95% = 1.09–2.16, *p* = 0.001), nephrotoxic AKI (OR = 0.79, CI 95% = 0.61–0.93, *p* = 0.02), mechanical ventilation (OR = 1.49, CI 95% = 1.14–2.97, *p* < 0.001), and positive FB after two PD sessions (OR = 1.08, CI 95% = 1.03–1.91, *p* = 0.007) were associated significantly with death, as shown in Table 5.

**Table 5:** Association (with *p* < 0.25) between multiple adjusted patient and peritoneal dialysis characteristics and death.

**Variables**	**OR (CI 95%)**	***P***
Age (per 1 year)	1.02 (0.98–1.06)	0.18
Nephrotoxic ATN	0.85 (0.71–0.92)	0.02
Mechanical ventilation	1.17 (1.09–2.99)	0.03
MELD	1.35 (1.27–4.11)	0.01
^*^Positive FB (per 1 l/day)	1.49 (1.34–3.87)	0.03

*ATN, acute tubular necrosis; FB, fluid balance (from 2nd to 4th peritoneal dialysis session); FB, fluid balance (l/day after the second HVPD session).*

* *After 2 dialysis session*.

## Discussion

The interest in PD for treatment of AKI patients has increased, and PD is actually used in developing countries because of its lower cost and minimal infrastructural requirements. Studies from these countries have shown that, with careful thought and planning, critically ill patients can be successfully treated using PD, achieving adequate metabolic and fluid control, and a mortality rate around 50% (13, 16–19). Recently, the pandemic of SARS-CoV-2 infection has overwhelmed HD capacity worldwide and acute PD has been an excellent alternative in developed countries as well (22–25).

In this study, we evaluated the role of PD in treating ACLF patients with AKI. Concerning infectious complications, the rate of peritonitis was similar to that reported in the literature (11–15%). Most of the patients who had their catheter removed and dialysis method changed underwent this due to lack of success with the treatment. The main mechanical complication was leakage and complications were less frequent than those reported in previous studies (16–19). We believe mechanical complications were less frequent because patients had ascites fluid, which made the catheter implantation technique easier, decreasing the migration of the catheter tips. Leakage was the most frequent (75%) mechanical complication, although it did not lead to interruption of therapy. The dialysate volume per cycle was reduced from 30 to 20 mL/kg per cycle (around 1,200 mL/cycle) and PD treatment was performed successfully.

In this series, both the in-hospital mortality rate and recovery of kidney function were worse than those found by previous studies that used PD for treating AKI patients, mainly cardiorrenal syndrome (CRS) type 1 and septic patients (13, 16–19, 26), and similar to other studies that treated cirrhotic patients by HD and CRRT (8, 10, 11).

In the first situation, we believe these differences occurred because ACLF patients have more severe disease than CRS type 1 or septic non-cirrhotic patients. Unfortunately, there are no controlled studies evaluating the indications, choice of dialysis methods, and effectiveness of dialysis in ACLF patients and the benefits of KRT remain controversial.

According to the literature, KRT should be considered for severe AKI, particularly for those patients on the waiting list for liver transplantation. The indications for KRT are not specific for patients with cirrhosis, and include uremia, volume overload, severe hyperkalemia, and severe metabolic acidosis, as described in our study. In patients with HRS, KRT should be indicated in the absence of response or adverse reaction to vasoconstrictors. Currently, both kidney and liver support in clinical studies did not show any survival advantage (11, 27, 28). The assessment of prognosis, eligibility for liver transplantation, and stage of ACLF should be considered before KRT to avoid futile treatments.

The choice of the dialytic method in decompensated cirrhosis or ACLF patients is critical and difficult to make. Worsening of circulatory dysfunction (i.e., severe arterial hypotension) during KRT is a major concern as it may cause organ failure. RRT is particularly poorly tolerated in patients with HRS, due to the profound haemodynamic disturbances that are characteristic of this syndrome.

Acceptable KRT methods are IHD, PHD, CVVT, and PD. The most important limiting factor of intermittent therapies is haemodynamic instability. Hypotension during KRT is associated with dialysis technique (volume and ultrafiltration rate, reduction of plasmatic osmolality) and patient characteristics (hypovolemia, vasodilation, liver failure) (5–8, 10, 11, 29, 30). Hypotension decreases the effectiveness of RRT and aggravates ischemic injury, delaying the recovery of kidney function. When compared to intermittent therapies, continuous methods offer greater haemodynamic stability, and are often preferred for patients with arterial hypotension (29–31).

Thrombocytopenia and coagulopathy limit the use of heparin or other anticoagulants during KRT. However, these coagulation disorders found in patients with cirrhosis do not protect patients against thrombosis during KRT (30). Regional citrate anticoagulation can be an alternative. However, citrate is metabolized by the liver and body clearance can be reduced in critically ill cirrhotic patients. In addition, citrate clearance cannot be predicted by standard liver function tests and serum ionized calcium level and blood pH should be monitored in haemodialysed cirrhotic patients.

Regarding the mode of dialysis, CRRT does not improve mortality in comparison with IHD; however, CRRT may be well-tolerated in patients with unstable conditions, including fulminant hepatic failure, as it does not raise intracranial pressure (28). Indeed, the ADQI group recommends KRT only in cases with acute reversible components.

In this scenario, PD may be better tolerated by cirrhotic patients than HD, with no increase in the number of complications, enabling removal of the ascites fluid, and not exposing patients to anticoagulants.

According to the *Recommendations of the Brazilian Society of Hepatology for the management of acute kidney injury in patients with cirrhosis*, PD should not be routinely used due to the increased risk of infections and mechanical complications. However, there is no previous study that has evaluated the use of PD in ACLF patients with AKI. The same groups also recommend the use of dialysis solutions with bicarbonate for patients with hyperlactatemia and regional anticoagulation (only in the dialysis circuit) for severe coagulopathy (5).

Our study showed encouraging results for BUN, creatinine, bicarbonate, and pH levels. Metabolic and fluid control were achieved after 3 or 4 PD sessions using continuous PD and prescribed Kt/V of 0.4. We prescribed a tidal modality to avoid the removal of all ascites fluid. Despite the poor lactic acid metabolism observed in patients with liver disease, we used lactate buffer and there was no increase in lactate levels and acidosis was corrected.

There was no significant difference between the survivors and non-survivors treated with PD in relation to metabolic control and delivered dialysis dose, which is consistent with previous studies on PD treatment for AKI (13, 16–19, 26).

Non-survivors had more severe clinical parameters and prognostic scores than survivors, such as higher age and MELD at hospital admission, and more patients needed mechanical ventilation. The two groups differed in the etiology of AKI, and FO after PD start. After two HVPD sessions, FB was significantly more negative in survivors than in non-survivors. Our results agree with previous studies, including systematic reviews, which have shown that FO is a risk factor and predictor of death in critical patients (21, 26, 32, 33).

In our study, positive FB was associated with higher mortality. Previous studies reported similar results. In a European multicenter trial, Payen et al. observed positive FB as a risk factor for mortality in 60 days in critical AKI patients (34). Wang et al. (21) in a multicenter ICU study showed the FB was greater in patients with AKI than in patients without AKI and that a higher cumulative FB was an important factor associated with 28-day mortality following AKI. A retrospective analysis of post-operative percentage FO in patients at AKI stage 3 after cardiac surgery showed that FO >7.2% was significantly associated with reduced 90-day survival [*p* < 0.001; (35)].

To the best our knowledge, this study is unique in providing detailed insights into PD treatment for ACLF patients and identifying risk factors for death.

Some limitations of this study must be considered. Firstly, the study was performed in a single center and the number of patients was small. Secondly, it was an observational study and PD treatment was not compared with another dialysis modality. Thirdly, it must also be recognized that FO is a feature of acute kidney disease of any cause and the lack of confirmatory investigations such as concurrent echocardiography or cardiac biomarker data for all out-patients is a limitation. Fourth, we did not perform a multivariate analysis for late mortality because we believe it was not appropriate for predicting survival given that only six deaths occurred. Finally, there are many factors that affect the prognosis of patients with ACLF. MELD, and FO are two of these factors, but we need to perform a propensity analysis to further explore this issue.

In conclusion, the findings of our study suggest that careful prescription may contribute to providing adequate treatment for most ACLF patients without contraindications for PD use, allowing adequate metabolic and fluid control, with no increase in the number of infectious or mechanical complications. MELD, mechanical complications, and FB were factors associated with mortality, while nephrotoxic AKI was a protective factor. Further studies are needed to better investigate the role of PD in ACLF patients with AKI, comparing it with other haemodialysis modalities in the treatment for these patients.

## Transparency Declaration

The lead authors (DP and AB) confirm that the manuscript is an honest, accurate, and transparent account of the study being reported; that no important aspects of the study have been omitted; and that any discrepancies from the study as originally planned (and, if relevant, registered) have been explained.

## Data Availability Statement

The raw data supporting the conclusions of this article will be made available by the authors, without undue reservation.

## Ethics Statement

The studies involving human participants were reviewed and approved by Botucatu Medical School. The patients/participants provided their written informed consent to participate in this study.

## Author Contributions

DP and AB made substantial contributions to conception and design, acquisition of data, and analysis and interpretation of data. EP, CK, and DP were involved in drafting the manuscript. WZ and DD revising it critically for important intellectual content. DP and AB gave final approval of the version to be published. All authors agreed to be accountable for all aspects of the work in ensuring that questions related to the accuracy or integrity of any part of the work are appropriately investigated and resolved.

## Conflict of Interest

The authors declare that the research was conducted in the absence of any commercial or financial relationships that could be construed as a potential conflict of interest.

## Publisher's Note

All claims expressed in this article are solely those of the authors and do not necessarily represent those of their affiliated organizations, or those of the publisher, the editors and the reviewers. Any product that may be evaluated in this article, or claim that may be made by its manufacturer, is not guaranteed or endorsed by the publisher.
